# Improving scattering layer through mixture of nanoporous spheres and nanoparticles in ZnO-based dye-sensitized solar cells

**DOI:** 10.1186/1556-276X-9-295

**Published:** 2014-06-11

**Authors:** Chohui Kim, Hongsik Choi, Jae Ik Kim, Sangheon Lee, Jinhyun Kim, Woojin Lee, Taehyun Hwang, Suji Kang, Taeho Moon, Byungwoo Park

**Affiliations:** 1 WCU Hybrid Materials Program, Department of Materials Science and Engineering, Research Institute of Advanced Materials, Seoul National University, Seoul 151-744, South Korea; 2 Department of Materials Science and Engineering, Dankook University, Chungnam, Cheonan 330-714, South Korea

**Keywords:** Dye-sensitized solar cell, ZnO photoelectrode, Light trapping, Nanoparticle, Nanoporous sphere

## Abstract

A scattering layer is utilized by mixing nanoporous spheres and nanoparticles in ZnO-based dye-sensitized solar cells. Hundred-nanometer-sized ZnO spheres consisting of approximately 35-nm-sized nanoparticles provide not only effective light scattering but also a large surface area. Furthermore, ZnO nanoparticles are added to the scattering layer to facilitate charge transport and increase the surface area as filling up large voids. The mixed scattering layer of nanoparticles and nanoporous spheres on top of the nanoparticle-based electrode (bilayer geometry) improves solar cell efficiency by enhancing both the short-circuit current (*J*_sc_) and fill factor (FF), compared to the layer consisting of only nanoparticles or nanoporous spheres.

## Background

Dye-sensitized solar cells (DSSCs) have shown promising potential as an alternative to Si thin-film solar cells because of low fabrication cost and relatively high efficiency [1,2]. Efficient utilization of sunlight is greatly important in photovoltaic systems for high efficiency. Therefore, there have been many studies on the scattering layer to fully utilize incident light inside solar cells by using different morphologies and sizes of scatterers in TiO_2_-based DSSCs [3-10]. However, few studies for the scattering layer exist in ZnO-based DSSCs [11-13], despite the advantages of ZnO such as higher carrier mobility and fabrication easiness for various nanostructures [14,15].

Among various nanostructures, hundred-nanometer-sized nanoporous spheres provide both effective light scattering and large surface area [16]. X. Tao's group and W. Que's group have reported on the scattering layer consisting of nanoporous spheres [17,18]. While they have shown improvements on the scattering effect, large voids between spheres leave the possibility of providing more available surface area where dye can be attached, and better charge transport by improved percolation of large-sized spheres should be achieved.

In this paper, we report the improvements of scattering layers using a mixture of nanoparticles and nanoporous spheres. Nanoporous spheres act as effective light scatterers with the large surface area, and nanoparticles favor both efficient charge transport and an additional surface area.

## Methods

The ZnO nanoporous spheres were synthesized by using zinc acetate dihydrate (0.01 M, Zn(CH_3_COO)_2_ · 2H_2_O, Sigma-Aldrich, St. Louis, MO, USA) and diethylene glycol ((HOCH_2_CH_2_)_2_O, Sigma-Aldrich) in an oil bath at 160°C for 6 h [16]. After washing with ethanol, the as-synthesized ZnO nanoporous spheres (NS) and ZnO nanoparticle (NP) (721085, Sigma-Aldrich) were mixed to the weight ratios of NP to NS of 10:0, 7:3, 5:5, 3:7, and 0:10. To fabricate bilayer-structured electrodes, a paste consisting of only ZnO nanoparticles (NP/NS = 10:0) was first spread on a fluorine-doped tin oxide substrate (FTO, TEC 8, Pilkington, St. Helens, UK) covered with a dense TiO_2_ blocking layer by sputtering. After solvent evaporation, the mixed pastes with various ratios of NS and NP were spread on top of the nanoparticle film by a doctor blade method. The active area was 0.28 cm^2^, and the as-deposited films were subsequently annealed at 350°C for 1 h.

The films were sensitized with 0.5 mM of N719 dye (RuL_2_(NCS)_2_:2TBA, L = 2,2′-bipyridyl-4,4′-dicarboxylic acid, TBA = tetrabutylammonium, Solaronix, Aubonne, Switzerland) for 30 min at RT. The sensitized electrode and platinized counter electrode were sealed with thermoplastic foil (25 μm, DuPont, Wilmington, DE, USA), and the gap between the two electrodes was filled with an iodide-based redox electrolyte (AN-50, Solaronix).

X-ray diffraction (XRD; M18XHF-SRA, Mac Science, Tokyo, Japan) was employed to analyze the crystal structure of the ZnO electrodes, and field emission scanning electron microscopy (FE-SEM; SU70, Hitachi, Tokyo, Japan) was used to observe the morphology of the bilayer-structured electrodes. The electrochemical properties were analyzed by a solar cell measurement system (K3000, McScience, Suwon, South Korea) under a solar simulator (xenon lamp, air mass (AM) 1.5, 100 mW cm^−2^). The extinction and diffused reflectance spectra were recorded on a UV/Vis spectrophotometer (Cary 5000, Agilent Technologies, Santa Clara, CA, USA), and incident photon-to-current conversion efficiency (IPCE) spectra were measured by an IPCE measurement system (K3100, McScience). Electrochemical impedance spectra (EIS) were taken by using a potentiostat (CHI 608C, CH Instrumental Inc., Austin, TX, USA) to analyze the kinetic parameters in the DSSCs [19-21].

## Results and discussion

**Figure 1:**
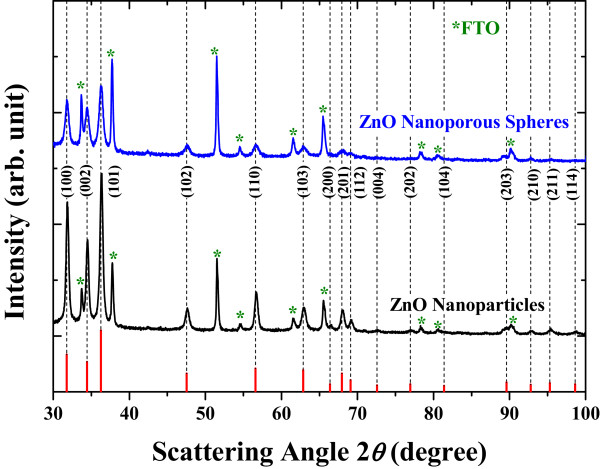
**X-ray diffraction of the ZnO films consisting of only nanoparticles or nanoporous spheres.** The peak intensities and positions from the hexagonal ZnO (JCPDS #36-1451) are shown as solid lines.

**Figure 2:**
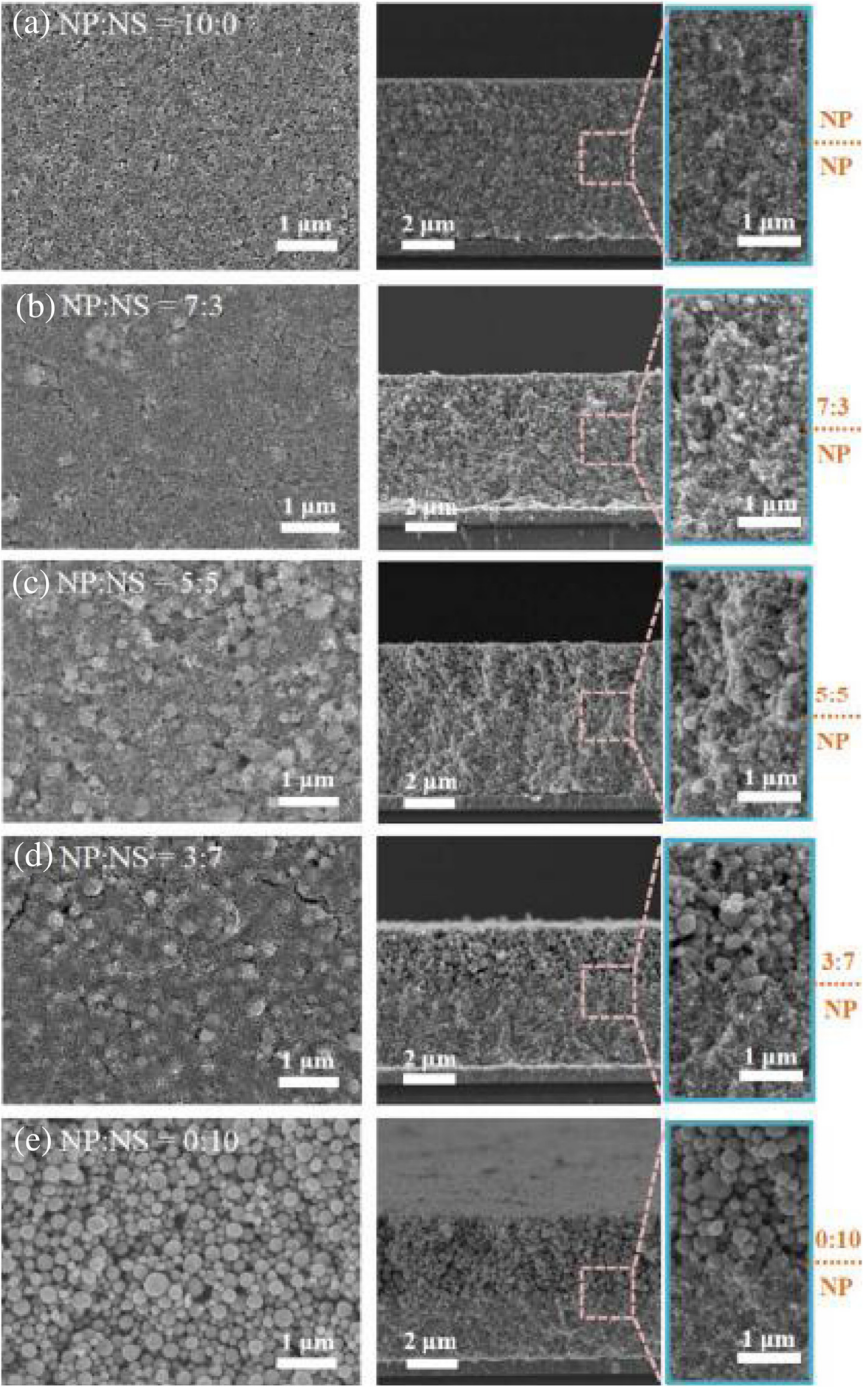
**Plan-view and cross-sectional SEM images of the ZnO bilayer electrodes.** The weight ratios of nanoparticle (NP) to nanoporous sphere (NS) for the top layers are **(a)** 10:0, **(b)** 7:3, **(c)** 5:5, **(d)** 3:7, and **(e)** 0:10, respectively. Blue labeled boxes are higher magnification for the bilayer interface.

The crystalline structure and grain size of ZnO nanoparticles and nanoporous spheres were analyzed by XRD (Figure 1). The diffraction confirms the crystalline ZnO having hexagonal wurtzite structure (JCPDS #36-1451). From Williamson-Hall plots [22-24], the homemade ZnO nanoporous spheres are composed of approximately 35-nm-sized grains, while the grain size of the commercial ZnO nanoparticles is approximately 55 nm.The ZnO bilayer electrodes were sequentially prepared by the bottom layer made by only ZnO nanoparticles and the top scattering layer formed with various mixing ratios of nanoparticles and nanoporous spheres. As shown in Figure 2, the plan-view SEM images of the scattering layers indicate that the nanoparticles and nanoporous spheres are mixed uniformly, not aggregated separately. The range of nanoporous sphere size is approximately 150 to 500 nm, with the average size of approximately 300 nm. As the ratio of nanoporous spheres increases, void spaces in the film get larger. The cross-sectional SEM images show that bilayer structures consisting of the nanoparticle bottom layer and mixed scattering upper layer are composed nicely without any crushes at the interface The average thickness of the bilayer films is approximately 5.5 μm, and the deviation is less than 10%. The poor connectivity among the ZnO nanoporous spheres with the decreased nanoparticle ratio is consistent with the plan-view SEM images.

**Figure 3:**
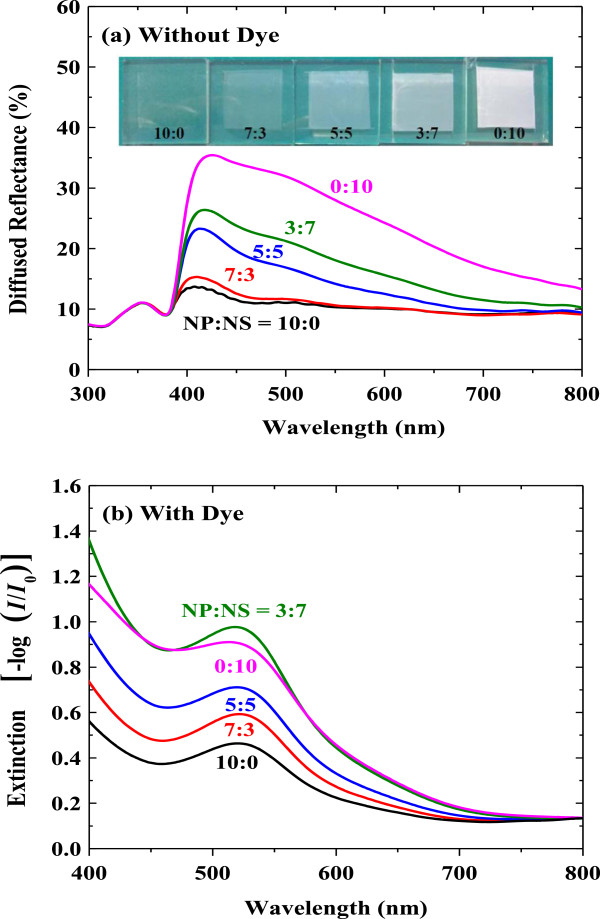
**Diffused reflectance and extinction spectra. (a)** Diffused reflectance spectra and optical images of the ZnO bilayer electrodes before dye loading with various mixing ratios. **(b)** Extinction spectra with dye loading.

To investigate the optical properties of the mixed scattering layer, the diffused reflectance of the bilayer films (without dye) was measured (Figure 3a) [25,26]. With the increased nanoporous sphere ratio, the diffused reflectance increases, indicating a better light scattering ability of nanoporous spheres due to the comparable size to the wavelength of visible light [27,28]. The optical images also confirm the scattering effect by the nanoporous spheres. When the ratio reaches to NP/NS = 0:10, the color changes to totally white.

Furthermore, after dye adsorption, the NP/NS = 3:7 film shows the highest extinction (Figure 3b). Especially when compared to the NP/NS = 0:10 film, the higher extinction near the dye absorption peak is clear [29]. The results indicate an optimum condition for the surface area between void filling by nanoparticles and primary nanoporous spheres. The notable change in the curve shape for the NP/NS = 0:10 film (Figure 3a,b) means that light scattering plays a role considerably for the adsorbed dye molecules [30].

**Figure 4:**
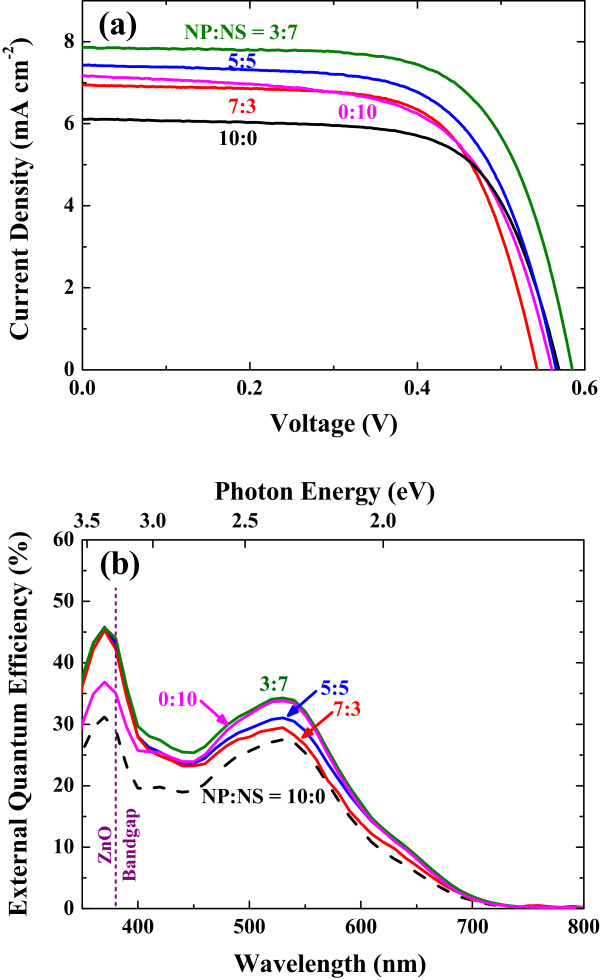
**Photocurrent-voltage curves and IPCE spectra. (a)** Photocurrent-voltage curves of the DSSCs with various mixing ratios. **(b)** Incident photon-to-current conversion efficiency (IPCE) spectra.

The solar cell performance of the DSSCs fabricated with the various ZnO bilayer electrodes was investigated (Figure 4a), and the parameters for each cell were summarized in Table 1 The mixed scattering layer improves both the short-circuit current (*J*_sc_) and fill factor (FF), compared to the nanoparticle layer. In particular, the optimum power conversion efficiency (*η*) of 2.91% is obtained at the ratio of NP/NS = 3:7, and the trend of *η* is generally consistent with that of *J*_sc_. The open-circuit voltage (*V*_oc_) values are not notably changed among the cells except for the NP/NS = 3:7. From the general trend of parameters, we cautiously consider that the value for the open-circuit voltage in NP/NS = 3:7 is out of the tendency. We consider different nanomorphologies of porous spheres synthesized from the limited number of samples. Open-circuit voltage is represented as $V_{\mathrm{oc}}\approx \frac{\mathrm{nkT}}{q}\cdot \ln\frac{J_{\mathrm{sc}}}{J_{0}}$ from the general one-diode model [31], and between the two conditions of the NP/NS = 5:5 and 3:7, the difference in *J*_sc_ (i.e., ln *J*_sc_) is not enough to impact *V*_oc_. Also, the change of *V*_oc_ may result from the difference of reverse saturation current *J*_0_. We have synthesized nanoporous ZnO spheres by hydrothermal method [16], and the nanostructural quality of porous ZnO spheres may vary from batch to batch, thus resulting in the difference of band offset, charge transfer mobilities, porosities, etc. [32,33].

**Table 1:** **Characteristics of photocurrent-voltage curves and charge transfer resistances (*R***_**ct**_**) for ZnO/electrolyte interfaces**

**NP/NS**	***J***_**sc **_**(mA cm**^**−2**^**)**	***V***_**oc **_**(V)**	**FF**	***η *** **(%)**	***R***_**ct **_**(Ω)**
10:0	5.98 ± 0.25	0.56 ± 0.01	0.67 ± 0.01	2.25 ± 0.15	30.7 ± 0.3
7:3	6.64 ± 0.30	0.55 ± 0.01	0.65 ± 0.02	2.36 ± 0.17	33.1 ± 0.2
5:5	7.45 ± 0.13	0.56 ± 0.01	0.68 ± 0.03	2.81 ± 0.14	29.8 ± 0.2
3:7	7.47 ± 0.24	0.58 ± 0.01	0.67 ± 0.01	2.91 ± 0.13	31.6 ± 0.2
0:10	7.28 ± 0.18	0.56 ± 0.01	0.64 ± 0.02	2.60 ± 0.09	34.5 ± 0.3

If charge collection probabilities are similar among the cells, quantum efficiency depends on the light trapping inside the solar cell [34-37]. The NP/NS = 3:7 cell exhibits the highest IPCE values in the whole visible region (Figure 4b), and this IPCE trend is consistent with the extinction data (Figure 3b). Therefore, the enhanced light-harvesting capability (i.e., *J*_sc_) by the mixed scattering layer is attributed to efficient light scattering and increased surface area.

**Figure 5:**
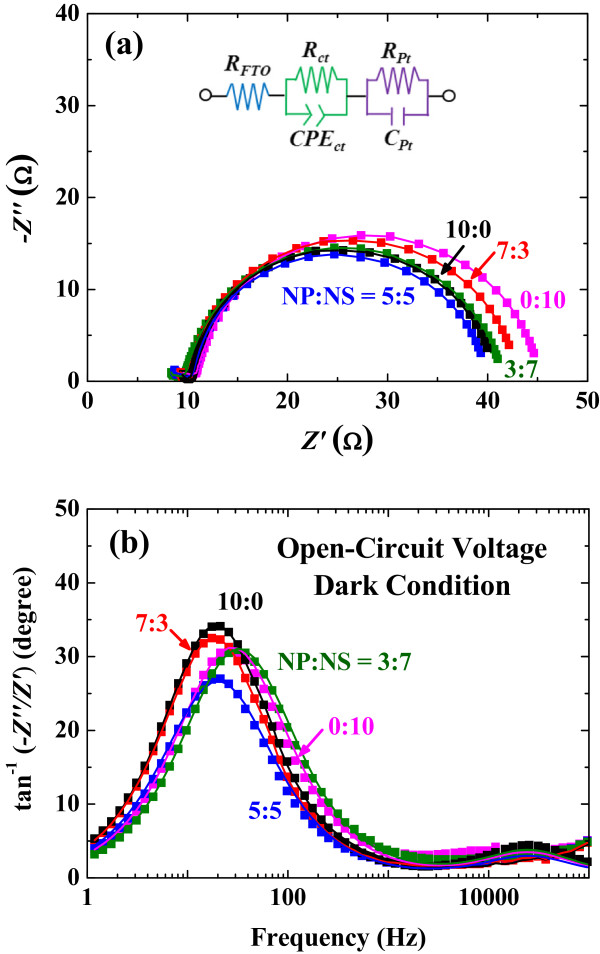
**Plots with various mixing ratios of ZnO nanoparticle to nanoporous sphere. (a)** Nyquist plot and **(b)** Bode plot. Solid lines are the fitting results using the equivalent circuit model in the inset.

Impedance analyses were performed to understand the electrical properties of the synthesized solar cells [38-41]. The Nyquist plots display two semicircles in Figure 5a; the larger semicircles in low frequency range (approximately 10^0^ to 10^3^ Hz) are related to the charge transport/accumulation at dye-attached ZnO/electrolyte interfaces, and the smaller semicircles in high frequency (approximately 10^3^ to 10^5^ Hz) are ascribed to the charge transfer at the interfaces of electrolyte/Pt counter electrode [42]. The impedance parameters were extracted using the equivalent circuit model (inset of Figure 5a), and the fitting lines are shown as solid lines in the Nyquist and Bode plots. From the charge transfer resistances (*R*_ct_) in Table 1, we can see that the proper mixing ratio (e.g., 5:5 or 3:7) exhibits lower values implying more efficient charge transfer processes across the ZnO/electrolyte interfaces, while the pure nanoporous sphere layer (0:10) shows the highest *R*_ct_. The low resistance favors the transport of the electrons injected within ZnO, thus eventually leading to an effective collection of electrons [11]. The better connectivity achieved by the nanoparticles likely facilitates charge transfer by providing electron transport pathways, thereby resulting in the enhancement of FF with less recombination.

## Conclusions

To improve the utilization of scattering layer in ZnO-based DSSCs, nanoparticles and nanoporous spheres are mixed with various ratios. The nanoporous spheres play an important role in the scattering effect with the large surface area but possess disadvantages of large voids and point contacts between spheres. Nanoparticles clearly advance facile carrier transport with the additional surface area, thereby improving the solar cell efficiency by the enhanced short-circuit current (*J*_sc_) and fill factor (FF).

## Competing interests

The authors declare that they have no competing interests.

## Authors' contributions

CK carried out the overall scientific experiment and drafted the manuscript. HC and JIK performed the FE-SEM measurements. SL carried out the analysis of electrochemical impedance spectra. JK and SK participated in the manuscript revision. WL and TH helped to check typing errors. BP and TM gave valuable advices about the whole experiments and manuscript as supervisors. All authors read and approved the final manuscript.
